# Molecular network profiling of U373MG human glioblastoma cells following induction of apoptosis by novel marine-derived anti-cancer 1,2,3,4-tetrahydroisoquinoline alkaloids

**DOI:** 10.1186/1475-2867-12-14

**Published:** 2012-04-11

**Authors:** Hiroko Tabunoki, Naoki Saito, Khanit Suwanborirux, Kornvika Charupant, Jun-ichi Satoh

**Affiliations:** 1 Department of Bioinformatics and Molecular Neuropathology, Meiji Pharmaceutical University, 2-522-1 Noshio, Kiyose, Tokyo, 204-8588, Japan; 2 Department of Pharmaceutical Chemistry, Graduate School of Pharmaceutical Sciences, Meiji Pharmaceutical University, 2-522-1 Noshio, Kiyose, Tokyo, 204-8588, Japan; 3 Center for Bioactive Natural Products from Marine Organisms and Endophytic Fungi (BNPME), Department of Pharmacognosy and Pharmaceutical Botany, Faculty of Pharmaceutical Sciences, Chulalongkorn University, Pathumwan, Bangkok, 10330, Thailand; 4 Bureau of Drug and Narcotic, Department of Medical Sciences, Ministry of Public Health, Tiwanond Road, Nonthaburi, 11000, Thailand

## Abstract

**Background:**

Glioblastoma is the most aggressive form of brain tumors showing resistance to treatment with various chemotherapeutic agents. The most effective way to eradicate glioblastoma requires the concurrent inhibition of multiple signaling pathways and target molecules involved in the progression of glioblastoma. Recently, we obtained a series of 1,2,3,4-tetrahydroisoquinoline alkaloids with potent anti-cancer activities, including ecteinascidin-770 (ET-770; the compound 1a) and renieramycin M (RM; the compound 2a) from Thai marine invertebrates, together with a 2’*-N*-4”-pyridinecarbonyl derivative of ET-770 (the compound 3). We attempted to characterize the molecular pathways responsible for cytotoxic effects of these compounds on a human glioblastoma cell line U373MG.

**Methods:**

We studied the genome-wide gene expression profile on microarrays and molecular networks by using pathway analysis tools of bioinformatics.

**Results:**

All of these compounds induced apoptosis of U373MG cells at nanomolar concentrations. The compound 3 reduced the expression of 417 genes and elevated the levels of 84 genes, while ET-770 downregulated 426 genes and upregulated 45 genes. RM decreased the expression of 274 genes and increased the expression of 9 genes. The set of 196 downregulated genes and 6 upregulated genes showed an overlap among all the compounds, suggesting an existence of the common pathways involved in induction of apoptosis. We identified the ErbB (EGFR) signaling pathway as one of the common pathways enriched in the set of downregulated genes, composed of PTK2, AKT3, and GSK3B serving as key molecules that regulate cell movement and the nervous system development. Furthermore, a GSK3B-specific inhibitor induced apoptosis of U373MG cells, supporting an anti-apoptotic role of GSK3B.

**Conclusion:**

Molecular network analysis is a useful approach not only to characterize the glioma-relevant pathways but also to identify the network-based effective drug targets.

## Background

Glioblastoma, World Health Organization (WHO) grade IV, is the most common and aggressive form of primary malignant brain tumors affecting the adult human brain. Glioblastoma is categorized into two distinct subtypes that develop through different genetic and molecular pathways, designated as primary glioblastoma and secondary glioblastoma [1]. Primary glioblastoma that develops without precursor lesions shows an amplification of the epidermal growth factor receptor (EGFR) gene more frequently than secondary glioblastoma that generates via the stepwise progression from the pre-existing low-grade glioma. EGFR amplification in primary glioblastoma is often associated with the expression of EGFRvIII, a ligand-independent constitutively active mutant of EGFR, capable of persistently activating the phosphatidylinositol 3-kinase (PI3K)/v-akt murine thymoma viral oncogene homolog (AKT) signaling pathway that promotes the survival of glioma cells [2]. The genetic mutations of phosphatase and tensin homolog (PTEN), a negative regulator of AKT signaling pathway, are more frequent found in primary glioblastoma. In contrast, secondary glioblastoma more often shows tumor protein p53 (TP53) mutations, and fairly consistently exhibits the genetic mutation of isocitrate dehydrogenase 1 (IDH1), a negative regulator of hypoxia-inducible factor 1-alpha (HIFA), whereas IDH1 mutations are barely detectable in primary glioblastoma [1].

Despite the multidisciplinary therapy that combines maximal surgical resection, radiation and chemotherapy, the median survival period does not usually exceed 2 years in the patients with glioblastoma owing to the high incidence of recurrence, invasion, and progression of the tumors [3]. The current drugs that specifically target the tyrosine kinase activity of EGFR or selectively inhibit the mammalian target of rapamycin (mTOR), a PI3K/AKT downstream signal transducer showed little efficacy in treatment of primary glioblastoma [3]. These observations suggest that the best way to eradicate glioblastoma requires the concurrent inhibition of multiple signaling pathways and target molecules that play a pivotal role in the survival and progression of glioma cells.

After the completion of the Human Genome Project, the global analysis of genome, transcriptome, proteome, and metabolome, collectively termed omics, promotes us to characterize the genome-wide molecular basis of the diseases, and helps us to identify disease-specific molecular signatures and biomarkers for diagnosis, classification, and prediction of prognosis. Because omics studies usually produce high-throughput experimental data at one time, it is often difficult to find out the meaningful biological implications from such a huge dataset. Recent advances in bioinformatics and systems biology have made major breakthroughs by illustrating the cell-wide map of complex molecular interactions with the aid of the literature-based knowledgebase of molecular pathways [4]. The logically arranged molecular networks construct the whole system characterized by robustness, which maintains the proper function of the system in the face of genetic and environmental perturbations. In the scale-free molecular networks, targeted disruption of limited numbers of critical components designated hubs, on which the biologically important molecular connections concentrate, could disturb the whole cellular function by destabilizing the networks [5]. Based on these views, the integration of omics data derived from glioma cells with underlying molecular networks provides a useful approach not only to characterize the glioma-relevant pathways but also to identify the network-based effective drug targets.

**Figure 1:**
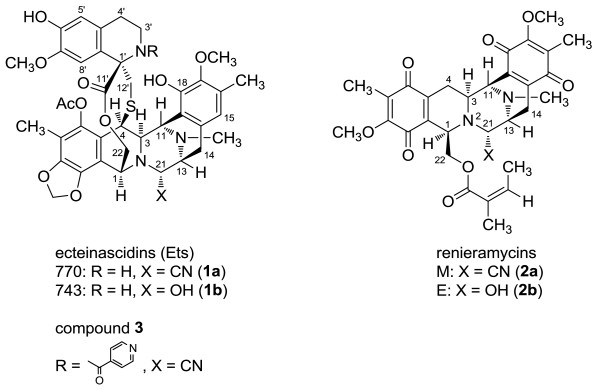
**The structures of novel 1,2,3,4-tetrahydroisoquinoline alkaloids.** Ecteinascidin-770 (ET-770, the compound **1a**), Ecteinascidin-743 (ET-743, the compound **1b**), renieramycin M (the compound **2a**), renieramycin E (the compound **2b**), and a 2’*-N*-4”-pyridinecarbonyl derivative of ET-770 (the compound **3**) are shown. The present study focused on cytotoxic effects of the compounds **1a**, **2a**, and **3**.

Various tetrahydroisoquinoline alkaloids with anti-cancer activities were isolated from marine invertebrates. They are classified into two major categories, ecteinascidins and renieramycins and their related compounds. The most potent bioactive member of ecteinascidins is ecteinascidin-743 (ET-743; the compound 1b in Figure 1, Trabectedin, Yondelis, CID 108150) isolated from the Carribean tunicate *Ecteinascidia turbinata*. The European Commission has approved ET-743 for the first-line therapy in the patients with unresectable soft tissue sarcoma refractory to the conventional chemotherapy [6]. ET-743 has a unique mechanism of anti-cancer activity in that it binds to the minor groove of the DNA double helix and alkylates the N2 of guanine to interfere with cell division, DNA repair, and transcriptional activation, leading to apoptosis of target cells [7-9].

Previously, we purified and identified a series of biologically active compounds from Thai marine invertebrates. Among them, ecteinascidin-770 (ET-770; the compound 1a in Figure 1, CID 16728498) is a stabilized derivative of ET-743 that maintained anti-cancer activities, obtained on a large scale from Thai tunicate *Ecteinascidia thurstoni* by pretreatment with potassium cyanide in buffer solution [10]. Recently, we prepared nitrogen-containing heterocyclic derivatives of ET-770, and we found that a 2’-*N*-4”-pyridinecarbonyl derivative of ET-770 (the compound 3 in Figure 1) shows the greater cytotoxicity to human colon, lung and prostate cancer cells than the compound 1a [11]. We also isolated renieramycin M (RM; the compound 2a in Figure 1), a major bis-1,2,3,4-tetrahydroisoquinolinequinone alkaloid from the Thai marine sponge *Xestospongia* sp*.*, which exhibits a potent cytotoxicity to human colon and breast cancer cells at the nanomolar concentrations [12]. Transcriptome analysis of the compound 2a-treated cancer cells suggested that the downregulation of protein tyrosine phosphatase receptor type K (PTPRK) serves as a biomarker for monitoring anti-tumor effects of RM [13]. Furthermore, RM induces apoptosis through the p53-dependent pathway and inhibits progression and metastasis of lung cancer cells at low sublethal concentrations [14].

The present study attempted to characterize and elucidate the molecular pathways responsible for cytotoxic effects of three distinct marine-derived anti-cancer 1,2,3,4-tetrahydroisoquinoline alkaloids 1a, 2a, and 3 on a human glioblastoma cell line U373MG by investigating the genome-wide gene expression profile and the relevant molecular networks.

## Methods

### Anti-cancer chemical compounds

The isolation, purification, chemical synthesis, and evaluation of cytotoxicity of renieramycin M (RM, the compound 2a), ecteinascidin-770 (ET-770, the compound 1a), and a 2’*-N*-4”-pyridinecarbonyl derivative of ET-770, the compound 3 were previously described in detail [10-15]. The chemical structures of these compounds are shown in Figure 1. For a stock solution, all of them are dissolved at the concentration of 10 mM in dimethyl sulfoxide (DMSO), and further diluted with culture medium at a working concentration prior to use. An equivalent concentration (v/v) of vehicle (DMSO) was included to serve as negative controls.

### Treatment of U373MG glioblastoma cells with anti-cancer chemical compounds

To determine the 50 % inhibitory concentration (IC50), U373MG human glioblastoma cells, incubated in Dulbecco’s Modified Eagle’s medium (DMEM; Invitrogen, Carlsbad, CA, USA) supplemented with 10 % fetal bovine serum (FBS), 100 U/ml penicillin and 100 μg/ml streptomycin (feeding medium), were exposed to the chemical compounds for varying periods at variable concentrations. Then, we assessed the cell viability by morphological observations and by using the 3-[4,5-dimethylthiazol-2-yl]-2,5-diphenyl tetrazolium bromide (MTT) cell growth kit (Millipore, Temecula, CA, USA). The cells were incubated for 4 to 72 hours in the feeding medium with inclusion of the chemical compounds at the IC50 concentration or the vehicle, and then were processed for western blot and microarray analysis. In some experiments, the cells were exposed for 36 hours to 20 μM glycogen synthase kinase 3-beta (GSK3B) inhibitor VII (EMD Chemicals, Gibbstown, NJ, USA).

### qPCR analysis

Total cellular RNA was extracted by using TRIZOL (Invitrogen). RNA treated with DNase I was processed for cDNA synthesis using oligo(dT)_20_ primers and SuperScript II reverse transcriptase (Invitrogen). For quantitative RT-PCR (qPCR) analysis, cDNA was amplified by PCR in LightCycler ST300 (Roche Diagnostics, Tokyo, Japan) using SYBR Green I and a panel of sense and antisense primer sets following: 5’ atgaccagcctccagcaagagtac3’ and 5’ agagggtagcaagacgtgctccta3’ for an 167 bp product of PTK2 protein tyrosine kinase 2 (PTK2); 5’cagatgtctccagtggactactgt3’ and 5’gttgtagaggcatccatctcttcc3’ for an 192 bp product of v-akt murine thymoma viral oncogene homolog 3 (AKT3); 5’gtaatccacctctggctaccatcc3’ and 5’aggtggagttggaagctgatgcag3’ for an 156 bp product of GSK3B; 5’gttgcagtcttgcgtgtggatgg3’ and 5’ggtgaccatgggaagcccatttg3’ for an 190 bp product of cell division cycle 25 homolog A (CDC25A); and 5’ccatgttcgtcatgggtgtgaacca3’ and 5’gccagtagaggcagggatgatgttc3’ for a 251 bp product of the glyceraldehyde-3-phosphate dehydrogenase (G3PDH) gene. The expression levels of target genes were standardized against the levels of G3PDH, an internal control, detected in the corresponding cDNA samples. All the assays were performed in triplicate.

### Microarray analysis

For microarray analysis, total cellular RNA was isolated by using the TRIZOL Plus RNA Purification kit (Invitrogen). The quality of total RNA was evaluated on Agilent 2100 Bioanalyzer (Agilent Technologies, Palo Alto, CA, USA). Three hundred ng of total RNA was processed for cRNA synthesis, fragmentation, and terminal labeling with the GeneChip Whole Transcript Sense Target Labeling and Control Reagents (Affymetrix, Santa Clara, CA, USA). Then, it was processed for hybridization at 45°C for 17 hours with Human Gene 1.0 ST Array (28,869 genes; Affymetrix). The arrays were washed in the GeneChip Fluidic Station 450 (Affymetrix), and scanned by the GeneChip Scanner 3000 7G (Affymetrix). The raw data were expressed as CEL files and normalized by the robust multiarray average (RMA) method with the Expression Console software (Affymetrix). Principal component analysis (PCA) of RMA-normalized data was performed on GeneSpring 11.5.1 (Agilent Technologies). All microarray data are available from the Gene Expression Omnibus (GEO) repository under the accession number GSE33619.

We performed three sets of the experiments independently, composed of the comparisons between the compound 1a and DMSO, between the compound 2a and DMSO, and between the compound 3 and DMSO. Each sample was processed individually for one array. Fold changes greater than 3 or smaller than 0.3333, calculated by the expression levels in the compound-treated cells divided by those in the vehicle-treated cells, were considered as substantial upregulation or downregulation.

### Molecular network analysis

The annotation of differentially expressed genes was studied by searching them on the Database for Annotation, Visualization and Integrated Discovery (DAVID) (david.abcc.ncifcrf.gov) [16]. To identify biologically relevant molecular networks of these genes, three distinct pathway analysis tools of bioinformatics endowed with comprehensive knowledgebase were used, including Kyoto Encyclopedia of Genes and Genomes (KEGG) (http://www.kegg.jp), Ingenuity Pathways Analysis (IPA) (Ingenuity Systems; http://www.ingenuity.com), and KeyMolnet (Institute of Medicinal Molecular Design; http://www.immd.co.jp).

KEGG includes manually curated reference pathways that cover a wide range of metabolic, genetic, environmental, and cellular processes, and human diseases. Currently, KEGG contains 158,816 distinct pathways generated from 417 reference pathways. By importing the list of Entrez Gene IDs into the Functional Annotation tool of DAVID, it identifies KEGG pathways and Gene Ontology (GO) categories composed of the genes enriched in the given set with statistical significance evaluated by the modified Fisher’s exact test. We have adjusted the EASE score threshold as 0.01 for the Functional Annotation tool of DAVID. The impact factor (IF) analysis that considers exact fold changes of differentially expressed genes for generation of the corresponding KEGG pathway was performed by using the Pathway-Express tool (Onto-Tools, Intelligent Systems and Bioinformatics Laboratory) [17].

IPA is a knowledgebase that contains approximately 2,500,000 biological and chemical interactions and functional annotations with definite scientific evidence, curated by expert biologists. By uploading the list of Gene IDs and expression values, the network-generation algorithm identifies focused genes integrated in a global molecular network. The network score, based on the hypergeometric distribution, is calculated by the right-tailed Fisher’s exact test not followed by multiple testing corrections. The network score represents the negative log of the p-value.

KeyMolnet contains knowledge-based contents on 137,300 relationships among human genes and proteins, small molecules, diseases, pathways and drugs, curated by expert biologists [4]. They are categorized into the core contents collected from selected review articles with the highest reliability or the secondary contents extracted from abstracts of PubMed and Human Reference Protein database (HPRD). By importing the list of Entrez Gene IDs, KeyMolnet automatically provides corresponding molecules as a node on networks. The neighboring network-search algorithm selects one or more molecules as starting points to generate the network of all kinds of molecular interactions around starting molecules, including direct activation/inactivation, transcriptional activation/repression, and the complex formation within the designated number of paths from starting points. The generated network was compared side by side with 459 human canonical pathways installed in the KeyMolnet library. The algorithm counting the number of overlapping molecular relations between the extracted network and the canonical pathway makes it possible to identify the canonical pathway showing the statistically significant contribution to the extracted network. The network-searching algorithm operating on KeyMolnet was described previously [4].

### Western blot analysis

To prepare total protein extract, the cells were homogenized in RIPA buffer supplemented with a cocktail of protease inhibitors (Sigma, St. Louis, MO, USA). The protein extract was centrifuged at 12,000 rpm for 5 min at room temperature (RT). The protein concentration was determined by a Bradford assay kit (BioRad Hercules, CA, USA). The mixture of the supernatant and a 2X Lammeli loading buffer was boiled and separated on a 12% SDS-PAGE gel. After gel electrophoresis, the protein was transferred onto nitrocellulose membranes, and immunolabeled at RT overnight with rabbit anti-poly-ADP-ribose-polymerase (PARP) antibody (#11835238001; Roche Diagnostics) or rabbit anti-cleaved caspase-3 (CASP3, Asp175) antibody (#9661; Cell Signaling Technology, Danvers, MA, USA). Then, the membranes were incubated at RT for 60 min with HRP-conjugated anti-rabbit IgG (Santa Cruz Biotechnology, Santa Cruz, CA, USA). The specific reaction was visualized by exposing the membranes to a chemiluminescent substrate (Thermo Scientific, Rockford, IL, USA). Then, the antibodies were stripped by incubating the membranes at 50°C for 30 min in stripping buffer, composed of 62.5 mM Tris–HCl, pH 6.7, 2% SDS and 100 mM 2-mercaptoethanol. The membranes were processed for relabeling with goat anti-heat shock protein HSP60 antibody (sc-1052, N-20; Santa Cruz Biotechnology) used for an internal control of protein loading, followed by incubation with HRP-conjugated anti-goat IgG (Santa Cruz Biotechnology).

## Results

### Induction of apoptosis of U373MG cells by exposure to the anti-cancer chemical compounds

**Figure 2:**
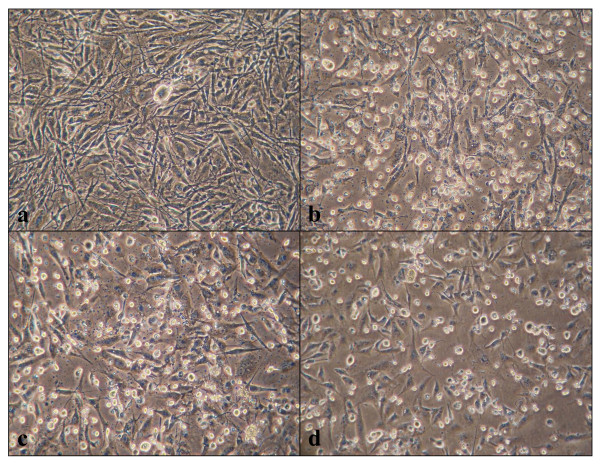
**Induction of apoptosis of U373MG cells by exposure to the anti-cancer chemical compounds.** U373MG cells were exposed for 72 hours to the chemical compounds individually at the concentration of IC_50_. The panels **(a-d)** represent phase contrast photographs of **(a)** the vehicle (DMSO), or **(b)** ET-770, the compound **1a**, **(c)** ET-770 derivative, the compound **3**, and **(d)** renieramycin M (RM), the compound **2a**. The round-shaped cells with remarkable shrinkage indicate the cells with ongoing apoptosis.

**Figure 3:**
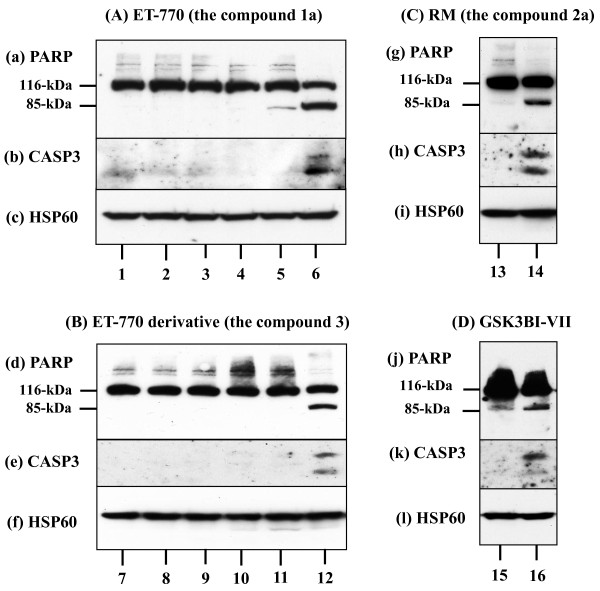
**Detection of apoptosis of U373MG cells by exposure to the anti-cancer chemical compounds.** U373MG cells were exposed to the chemical compounds individually at the concentration of IC_50_ for 72 hours or 20 μM GSK3B inhibitor VII for 36 hours. They were then processed for western blot analysis. The panels **(A-D)** represent the blot of **(A)** ET-770, the compound **1a**, **(B)** ET-770 derivative, the compound **3**, **(C)** RM, the compound **2a**, and **(D)** GSK3B inhibitor VII. The panels **(a-l)** indicate the blot of **(a, d, g, j)** poly-ADP-ribose-polymerase (PARP) composed of an 116-kDa uncleaved product and an 85-kDa cleaved product, **(b, e, h, k)** caspase-3 (CASP3) composed of 19-kDa and 17-kDa cleaved products, and **(c, f, i, l)** heat shock protein HSP60, an internal control of protein loading. The lanes **(1–16)** represent the cells **(1, 7)** untreated or treated for **(2, 8)** 4 hours, **(3, 9)** 8 hours, **(4, 10)** 24 hours, **(16)** 36 hours, **(5, 11)** 48 hours, and **(6, 12, 14)** 72 hours with the compounds or **(13)** 72 hours with the vehicle (DMSO), and **(15)** 36 hours with the vehicle (DMSO).

First of all, we determined the IC_50_ concentration of the compounds 1a, 2a, and 3 for killing U373MG glioblastoma cells in culture by using the MTT assay. We identified IC_50_ as 4.83 nM for the compound 1a, 3.10 nM for the compound 2a, and 1.70 nM for the compound 3 by a 72 hour-treatment, indicating that very low concentrations of the chemical compounds are sufficient to induce cell death of U373MG (Figure 2, panels b-d). The exposure of U373MG cells for 72 hours to each chemical compound at the concentration of IC_50_ induced the cleavage of PARP and CASP3, both of which reflect molecular markers of ongoing apoptosis (Figure 3, panels a, b, d, e, g, h, lanes 6, 12, 14).

### Gene expression profile of U373MG cells following exposure to the anti-cancer chemical compounds

Next, by using Human Gene 1.0 ST Array, we studied the genome-wide gene expression profile of U373MG cells exposed for 24 hours to the vehicle (DMSO) or to the compounds 1a, 2a, or 3 individually at the concentration of IC_50_. We performed three sets of the experiments independently, composed of the comparative analysis between the compound 1a and DMSO, between the compound 2a and DMSO, and between the compound 3 and DMSO. Principal component analysis (PCA) showed that the microarray data derived from the chemical compound-treated cells and those from DMSO-treated cells constituted two spatially-separated planes, suggesting that the treatment with the chemical compounds exhibits the much greater impact on gene expression profile than the levels of gene expression changes simply attributable to the technical errors (Additional file 1 online). Therefore, we considered the fold changes greater than 3 or smaller than 0.3333, calculated by the expression levels in the compound-treated cells divided by those in the vehicle-treated cells, as substantial upregulation or downregulation.

**Figure 4:**
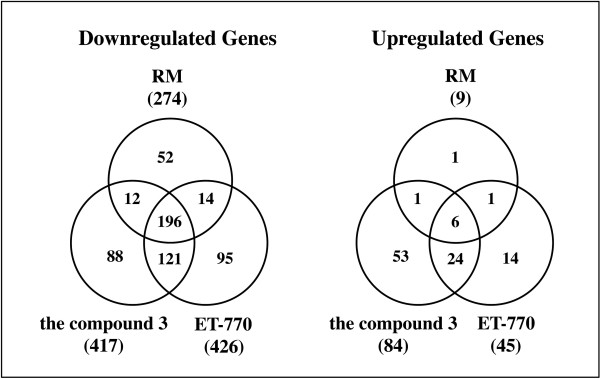
**The genes downregulated or upregulated in U373MG cells following exposure to the anti-cancer chemical compounds.** The genome-wide gene expression profile was studied in U373MG cells exposed for 24 hours to the vehicle (DMSO) or to the chemical compounds **1a**, **2a**, or **3** individually at the concentration of IC_50_. The fold changes greater than 3 or smaller than 0.3333, calculated by the expression levels in the compound-treated cells divided by those in the vehicle-treated cells, were considered as substantial upregulation or downregulation. The number of downregulated or upregulated genes is shown.

We identified a battery of downregulated genes and upregulated genes following treatment with each chemical compound (Figure 4). The compound 3 reduced the expression of 417 genes and elevated the levels of 84 genes, while the compound 1a downregulated 426 genes and upregulated 45 genes. The compound 2a decreased the expression of 274 genes and increased the expression of 9 genes. Overall, downregulated genes greatly outnumbered upregulated genes in each chemical compound. Top 10 genes downregulated or upregulated in U373MG are listed in Table 1. The complete list of all differentially expressed genes is available online as Additional files: Tables S1, S2, S3, S4, S5 and  S6.

**Table 1:** Top 10 genes downregulated or upregulated in U373MG cells following exposure to anti-cancer chemical compounds

**ET-770 (the Compound 1a)**				**ET-770 Derivative (the Compound 3)**				**RM (the Compound 2a)**			
**Downregulated Genes (n = 426)**				**Downregulated Genes (n = 417)**				**Downregulated Genes (n = 274)**			
**Rank **	**Gene Symbol**	**Gene Name**	**Fold Change**	**Rank **	**Gene Symbol**	**Gene Name**	**Fold Change**	**Rank **	**Gene Symbol**	**Gene Name**	**Fold Change**
1	SSBP2	single-stranded DNA binding protein 2	0.040	1	PTPRZ1	protein tyrosine phosphatase, receptor-type, Z polypeptide 1	0.043	1	PSD3	pleckstrin and Sec7 domain containing 3	0.068
2	ELMO1	engulfment and cell motility 1	0.049	2	SSBP2	single-stranded DNA binding protein 2	0.053	2	MST131	MSTP131	0.099
3	ODZ2	odz, odd Oz/ten-m homolog 2 (Drosophila)	0.053	3	NLGN1	neuroligin 1	0.055	3	PLCB1	phospholipase C, beta 1 (phosphoinositide-specific)	0.106
4	PRKD1	protein kinase D1	0.068	4	CD180	CD180 molecule	0.068	4	EXOC6B	exocyst complex component 6B	0.114
5	NLGN1	neuroligin 1	0.072	5	EPHA3	EPH receptor A3	0.073	5	ELMO1	engulfment and cell motility 1	0.125
6	MLLT3	myeloid/lymphoid or mixed-lineage leukemia (trithorax homolog, Drosophila); translocated to, 3	0.072	6	PRKD1	protein kinase D1	0.075	6	DPYD	dihydropyrimidine dehydrogenase	0.125
7	SBF2	SET binding factor 2	0.088	7	PSD3	pleckstrin and Sec7 domain containing 3	0.080	7	DIAPH2	diaphanous homolog 2 (Drosophila)	0.128
8	EPHA3	EPH receptor A3	0.089	8	ELMO1	engulfment and cell motility 1	0.084	8	PDE1C	phosphodiesterase 1 C, calmodulin-dependent 70 kDa	0.133
9	PSD3	pleckstrin and Sec7 domain containing 3	0.089	9	ODZ2	odz, odd Oz/ten-m homolog 2 (Drosophila)	0.084	9	FAM172A	family with sequence similarity 172, member A	0.138
10	ERC1	ELKS/RAB6-interacting/CAST family member 1	0.094	10	CASK	calcium/calmodulin-dependent serine protein kinase (MAGUK family)	0.094	10	FRMD5	FERM domain containing 5	0.139
**Upregulated Genes (n = 45)**				**Upregulated Genes (n = 84)**				**Upregulated Genes (n = 9)**			
**Rank**	**Gene Symbol**	**Gene Name**	**Fold Change**	**Rank**	**Gene Symbol**	**Gene Name**	**Fold Change**	**Rank**	**Gene Symbol**	**Gene Name**	**Fold Change**
1	OSR2	odd-skipped related 2 (Drosophila)	6.10	1	AKAP5	A kinase (PRKA) anchor protein 5	6.42	1	DENND2C	DENN/MADD domain containing 2 C	4.28
2	HBEGF	heparin-binding EGF-like growth factor	5.12	2	SLC25A19	solute carrier family 25 (mitochondrial thiamine pyrophosphate carrier), member 19	4.90	2	ZNF844	zinc finger protein 844	4.07
3	AKAP5	A kinase (PRKA) anchor protein 5	4.89	3	OSR2	odd-skipped related 2 (Drosophila)	4.86	3	HBEGF	heparin-binding EGF-like growth factor	4.02
4	CSNK1G1	casein kinase 1, gamma 1	4.69	4	ZNF844	zinc finger protein 844	4.79	4	OSR2	odd-skipped related 2 (Drosophila)	3.52
5	IL1A	interleukin 1, alpha	4.66	5	HBEGF	heparin-binding EGF-like growth factor	4.63	5	LRRTM2	leucine rich repeat transmembrane neuronal 2	3.44
6	GABRQ	gamma-aminobutyric acid (GABA) receptor, theta	4.28	6	CDC25A	cell division cycle 25 homolog A (S. pombe)	4.38	6	TSPYL2	TSPY-like 2	3.28
7	ZNF844	zinc finger protein844	4.03	7	ZNF684	zinc finger protein 684	4.24	7	TLCD1	TLC domain containing 1	3.10
8	E2F2	E2F transcription factor 2	3.82	8	TLCD1	TLC domain containing 1	4.10	8	RHEBL1	Ras homolog enriched in brain like 1	3.08
9	IL17RB	interleukin 17 receptor B	3.79	9	GPATCH4	G patch domain containing 4	4.06	9	GABRQ	gamma-aminobutyric acid (GABA) receptor, theta	3.00
10	B7H6	B7 homolog 6	3.75	10	CHAC2	ChaC, cation transport regulator homolog 2 (E. coli)	4.01				

The genome-wide gene expression profile was studied on Human Gene 1.0 ST array in U373MG cells following a 24 hour-exposure to the vehicle (DMSO) or to the three chemical compounds (Figure 1) individually at the concentration of IC_50_. The fold changes greater than 3 or smaller than 0.3333, calculated by the expression levels in the compound-treated cells divided by those in the vehicle-treated cells, were considered as substantial upregulation or downregulation. Top ten of them are listed with gene symbol, gene name, and fold change.

Importantly, 196 downregulated genes and 6 upregulated genes showed an overlap among three distinct compounds (all were listed in Additional file 8: Table S 7), suggesting an existence of the common molecular pathways involved in apoptosis induced by these chemical compounds. It is worthy to note that engulfment and cell motility 1 (ELMO1) and pleckstrin and Sec7 domain containing 3 (PSD3) were identified in the list of downregulated genes, while odd-skipped related 2 (OSR2), heparin-binding EGF-like growth factor (HBEGF), and zinc finger protein 844 (ZNF844) were included in upregulated genes shared among three chemical compounds (Table 1).

**Figure 5:**
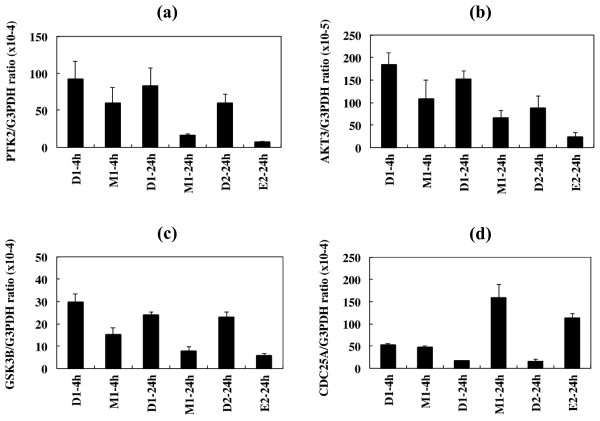
**qPCR of PTK2, AKT3, GSK3B, and CDC25A mRNAs in U373MG cells following exposure to the anti-cancer chemical compounds.** The panels **(a-d)** indicate quantitative RT-PCR (qPCR) analysis of **(a)** PTK2, **(b)** AKT3, **(c)** GSK3B, and **(d)** CDC25A mRNA expression standardized against the levels of G3PDH in U373MG cells exposed for 4 hours or 24 hours to DMSO **(D1)** or ET-770 derivative, the compound **3 (M1)**, or for 24 hours to DMSO **(D2)** or ET-770, the compound **1a (E2),** individually at the concentration of IC_50_.

Supporting the microarray analysis data, qPCR analysis validated substantial downregulation of PTK2, AKT3, and GSK3B and notable upregulation of CDC25A in U373MG cells following exposure to the compounds 3 and 1a (Figure 5).

### Molecular network of the genes altered in U373MG cells by the anti-cancer chemical compounds

**Figure 6:**
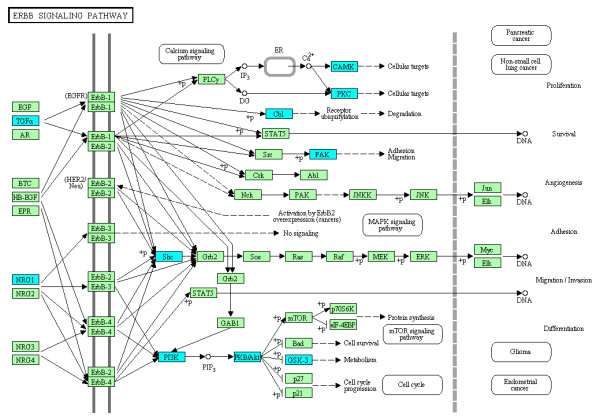
**Molecular network of downregulated genes in U373MG cells following exposure to ET-770, the compound 1a.** Entrez Gene IDs of 426 downregulated genes in U373MG cells following a 24 hour-exposure to the compound **1a** were imported into the Functional Annotation tool of DAVID. The KEGG pathways closely related to the set of imported genes were identified. The ErbB (EGFR) signaling pathway (hsa04012), identified as the first-rank downregulated pathway (Table 2), is shown, where downregulated genes are colored by blue.

**Figure 7:**
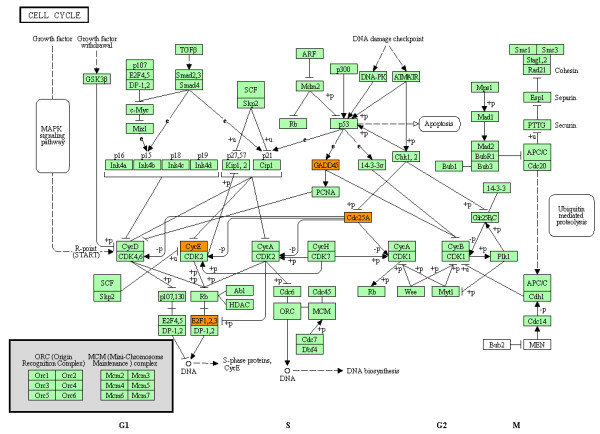
**Molecular network of upregulated genes in U373MG cells following exposure to ET-770 derivative, the compound 3.** Entrez Gene IDs of 84 upregulated genes in U373MG cells following a 24 hour-exposure to the compound **3** were imported into the Functional Annotation tool of DAVID. The KEGG pathways closely related to the set of imported genes were identified. The cell cycle pathway (hsa04110), identified as the first-rank upregulated pathway (Table 2), is shown, where upregulated genes are colored by orange.

By importing the complete list of Entrez Gene IDs of either downregulated or upregulated genes in U373MG cells after a 24 hour-exposure to the three compounds into the Functional Annotation tool of DAVID, we identified the KEGG pathways closely related to the set of imported genes (Table 2). We found that downregulated genes in U373MG cells shared among the three compounds showed the significant relevance to the ErbB (EGFR) signaling pathway (hsa04012), composed of focal adhesion kinase (FAK), alternatively named protein tyrosine kinase 2 (PTK2), v-akt murine thymoma viral oncogene homolog (AKT)/protein kinase B (PKB), and glycogen synthase kinase 3 (GSK3) acting as central signaling molecules (colored by blue in Figure 6), in addition to the axon guidance pathway (hsa04360). We also validated these findings by the Protein Analysis Through Evolutionary Relationships (PANTHER) classification system (http://www.pantherdb.org), operating on the different computational algorithms from KEGG (Additional file 9 online). The pathways of adherens junction (hsa04520), focal adhesion (hsa04510), and cell adhesion molecules (hsa04514) were also enriched in the set of downregulated genes by these chemical compounds (Table 2). In contrast, the set of upregulated genes by both the compounds 1a and 3 showed the most significant relationship with the cell cycle pathway (hsa04110) (colored by orange in Figure 7), where CDC25A serves as a hub molecule.

**Table 2:** KEGG pathways of the genes downregulated or upregulated in U373MG cells following exposure to anti-cancer chemical compounds

**ET-770 (the Compound 1a)**				**ET-770 Derivative (the Compound 3)**				**RM (the Compound 2a)**			
**Downregulated Genes (n = 426)**				**Downregulated Genes (n = 417)**				**Downregulated Genes (n =274)**			
**Rank**	**Pathway**	**P-Value**	**FDR**	**Rank**	**Pathway**	**P-Value**	**FDR**	**Rank**	**Pathway**	**P-Value**	**FDR**
1	hsa04012:ErbB signaling pathway	0.0006	0.71	1	hsa04510:Focal adhesion	0.0001	0.12	1	hsa04070:Phosphatidylinositol signaling system	0.0003	0.38
2	hsa04360:Axon guidance	0.0008	0.94	2	hsa04360:Axon guidance	0.0002	0.21	2	hsa04916:Melanogenesis	0.0019	2.17
3	hsa04070:Phosphatidylinositol signaling system	0.0041	4.69	3	hsa04810:Regulation of actin cytoskeleton	0.0007	0.80	3	hsa05223:Non-small cell lung cancer	0.0027	3.03
4	hsa04520:Adherens junction	0.0052	5.82	4	hsa04020:Calcium signaling pathway	0.0083	9.21	4	hsa04510:Focal adhesion	0.0032	3.51
5	hsa05214:Glioma	0.0076	8.42	5	hsa04012:ErbB signaling pathway	0.0093	10.22	5	hsa04020:Calcium signaling pathway	0.0043	4.69
				6	hsa04514:Cell adhesion molecules (CAMs)	0.0097	10.63	6	hsa04012:ErbB signaling pathway	0.0046	5.10
								7	hsa05214:Glioma	0.0053	5.86
								8	hsa04720:Long-term potentiation	0.0074	8.01
								9	hsa04912:GnRH signaling pathway	0.0083	8.91
								10	hsa04360:Axon guidance	0.0084	9.08
**Upregulated Genes (n = 45)**				**Upregulated Genes (n = 84)**				**Upregulated Genes (n = 9)**			
**Rank**	**Pathway**	**P-Value**	**FDR**	**Rank**	**Pathway**	**P-Value**	**FDR**	**Rank**	**Pathway**	**P-Value**	
1	hsa04110:Cell cycle	0.0053	4.58	1	hsa04110:Cell cycle	0.0077	6.84		none		

The genome-wide gene expression profile was studied on Human Gene 1.0 ST array in U373MG cells following a 24 hour-exposure to the vehicle (DMSO) or to the three chemical compounds (Figure 1). By importing the complete list of Entrez Gene IDs of downregulated or upregulated genes into the Functional Annotation tool of DAVID, the KEGG pathways related to the set of imported genes showing p < 0.01 by a modified Fisher Exact test were identified. The pathways are listed with the category, p-value, and false discover rate (FDR %).

The impact factor (IF) analysis by the Pathway-Express tool indicated that adherence junction (hsa04520) (IF = 29.856) and phosphatidylinositol signaling system (hsa04070) (IF = 26.696) in the compound 1a, phosphatidylinositol signaling system (hsa04070) (IF = 25.067) and adherence junction (hsa04520) (IF =21.93) in the compound 3, and phosphatidylinositol signaling system (hsa04070) (IF = 27.965) and adherence junction (hsa04520) (IF =13.422) in the compound 2a represented the pathways showing the greatest impact on the set of downregulated genes by these compounds (Additional file 10).

**Figure 8:**
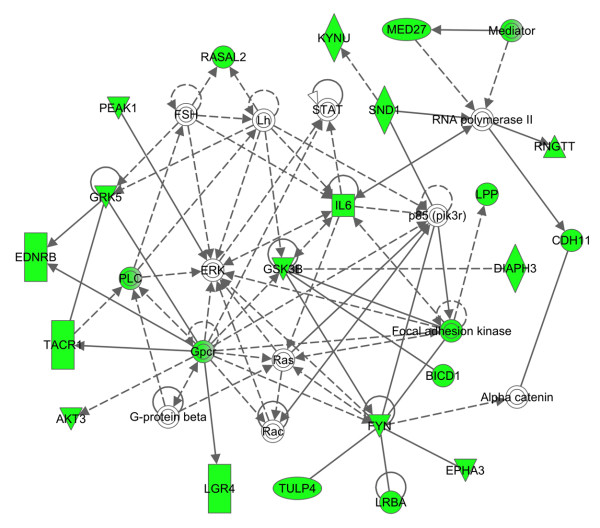
**Molecular network of downregulated genes in U373MG cells following exposure to renieramycin M, the compound 2a.** Entrez Gene IDs of 274 downregulated genes in U373MG cells following a 24 hour-exposure to the compound **2a** were imported into IPA. The molecular networks closely related to the set of imported genes were identified. The molecular network defined by “cellular movement, cell morphology, cell-to-cell signaling and interaction” identified as the third-rank downregulated network (Table 3) is shown, where downregulated genes are colored by green.

Next, by importing the complete list of Entrez Gene IDs of downregulated or upregulated genes in U373MG cells after a 24 hour-exposure to three compounds into IPA, we identified the molecular networks most closely related to the set of imported genes (Table 3). The molecular networks with functional categories defined by cellular movement and nervous system development and function were enriched in the network of the genes downregulated by all the three compounds (colored by green in Figure 8), where GSK3B acts as a hub molecule. In contrast, the molecular networks with functional categories defined by cellular growth and proliferation were overrepresented in the network of upregulated genes (Table 3).

**Table 3:** IPA networks of the genes downregulated or upregulated in U373MG cells following exposure to anti-cancer chemical compounds

**ET-770 (the Compound 1a)**			**ET-770 Derivative (the Compound 3)**			**RM (the Compound 2a)**		
**Downregulated Genes (n = 426)**			**Downregulated Genes (n = 417)**			**Downregulated Genes (n = 274)**		
**Rank**	**Associated Network Functions**	**P-Value**	**Rank**	**Associated Network Functions**	**P-Value**	**Rank**	**Associated Network Functions**	**P-Value**
1	Cellular Assembly and Organization, Cellular Function and Maintenance, Molecular Transport	1.00E-45	1	Cardiovascular Disease, Tissue Development, Post-Translational Modification	1.00E-43	1	Cellular Movement, Cardiac Arteriopathy, Cardiovascular Disease	1.00E-48
2	Tissue Development, Cellular Movement, Nervous System Development and Function	1.00E-36	2	Cell-To-Cell Signaling and Interaction, Cellular Assembly and Organization, Cellular Movement	1.00E-40	2	Gene Expression, Cellular Growth and Proliferation, Cell Death	1.00E-38
3	Developmental Disorder, Skeletal and Muscular Disorders, Nervous System Development and Function	1.00E-34	3	Cell-To-Cell Signaling and Interaction, Nervous System Development and Function, Cellular Function and Maintenance	1.00E-34	3	Cellular Movement, Cell Morphology, Cell-To-Cell Signaling and Interaction	1.00E-35
4	Gene Expression, Infectious Disease, Cellular Development	1.00E-34	4	Cellular Movement, Cellular Growth and Proliferation, Carbohydrate Metabolism	1.00E-31	4	Cellular Growth and Proliferation, Cellular Movement, Cell Death	1.00E-29
5	Post-Translational Modification, Cellular Assembly and Organization, Cellular Function and Maintenance	1.00E-28	5	Gene Expression, Cellular Development, Cellular Growth and Proliferation	1.00E-31	5	Cellular Movement, Nervous System Development and Function, Developmental Disorder	1.00E-28
**Upregulated Genes (n = 45)**			**Upregulated Genes (n = 84)**			**Upregulated Genes (n = 9)**		
**Rank**	**Associated Network Functions**	**P-Value**	**Rank**	**Associated Network Functions**	**P-Value**	**Rank**	**Associated Network Functions**	**P-Value**
1	Cell Cycle, Gene Expression, Cell Death	1.00E-41	1	Cellular Growth and Proliferation, Cell Cycle, Cell-To-Cell Signaling and Interaction	1.00E-39	1	Cellular Development, Cellular Growth and Proliferation, Cancer	1.00E-14
2	Inflammatory Response, Lipid Metabolism, Small Molecule Biochemistry	1.00E-27	2	Gene Expression, Infectious Disease, Cell-To-Cell Signaling and Interaction	1.00E-32	2	Cell-To-Cell Signaling and Interaction, Molecular Transport, Small Molecule Biochemistry	1.00E-03
3	Cellular Development, Cellular Growth and Proliferation, Respiratory System Development and Function	1.00E-14	3	Cellular Development, Cellular Growth and Proliferation, Endocrine System Development and Function	1.00E-21			
4	Cancer, Carbohydrate Metabolism, Endocrine System Disorders	1.00E-03	4	Cellular Movement, DNA Replication, Recombination, and Repair, Gene Expression	1.00E-11			
5	Cell Death, Post-Translational Modification, Amino Acid Metabolism	1.00E-03	5	Cellular Compromise, Cell Morphology, Inflammatory Response	1.00E-02			

The genome-wide gene expression profile was studied on Human Gene 1.0 ST array in U373MG cells following a 24 hour-exposure to the vehicle (DMSO) or to the three chemical compounds (Figure 1). By importing the complete list of Entrez Gene IDs of downregulated or upregulated genes into IPA, the molecular networks related to the set of imported genes were identified. The networks are listed with the functional category and p-value.

**Figure 9:**
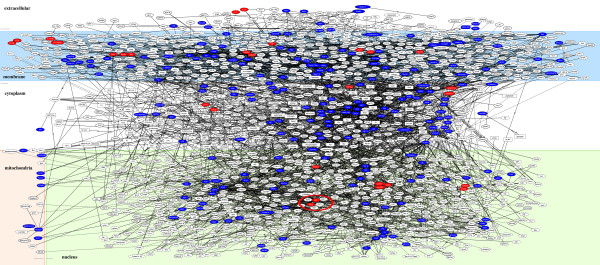
**Molecular network of downregulated and upregulated genes in U373MG cells following exposure to the compounds 1a and 3.** Entrez Gene IDs of the combined set of 317 downregulated genes and 30 upregulated genes in U373MG cells shared by the compounds **1a** and **3** were imported into KeyMolnet. The neighboring network-search algorithm on core contents illustrated the highly complex molecular network showing the most significant relationship with transcriptional regulation by Rb/E2F. Red or blue nodes represent upregulated or downregulated genes, respectively. White nodes exhibit the molecules extracted automatically from the core contents of KeyMolnet to establish molecular connections. The molecular relation is indicated by solid line with arrow (direct binding or activation), solid line with arrow and stop (direct inactivation), solid line without arrow (complex formation), dash line with arrow (transcriptional activation), and dash line with arrow and stop (transcriptional repression). The red circle indicates the E2F family of transcription factors.

Finally, the sets of 317 downregulated genes and 30 upregulated genes in U373MG cells shared between the structurally-related compounds 1a and 3 were combined together, and then the corresponding Entrez Gene IDs were imported into KeyMolnet. By using the neighboring network-search algorithm, we identified the highly complex network composed of 1588 molecules and 2171 molecular relations, showing the most significant relationship with the canonical pathway of transcriptional regulation by retinoblastoma protein (Rb)/E2F transcription factors (p = 2.202E-219) (Figure 9).

Because GSK3B acts as a central player in the molecular network of downregulated genes in U373MG cells by the three compounds, we determined the direct effect of a GSK3B inhibitor on the survival of U-373MG cells. Following a 36 hour-exposure to 20 μM GSK3B inhibitor VII, a cell-permeable and non-ATP competitive inhibitor with the high specificity for GSK3B, we found induction of apoptosis of U373MG cells, suggesting an anti-apoptotic role of GSK3B (Figure 3, panels j, k, lane 16).

## Discussion

To identify the molecular pathways responsible for cytotoxic effects of novel 1,2,3,4-tetrahydroisoquinoline alkaloids 1a, 2a, and 3 on U373MG cells, we studied the genome-wide gene expression profile and molecular networks by using pathway analysis tools of bioinformatics. All of these compounds induced apoptosis of U373MG cells at physiologically relevant nanomolar concentrations. The compound 3 reduced the expression of 417 genes and elevated the levels of 84 genes, while the compound 1a downregulated 426 genes and upregulated 45 genes. The compound 2a decreased the expression of 274 genes and increased the expression of 9 genes. Importantly, the set of 196 downregulated genes and 6 upregulated genes showed an overlap among all of these compounds, suggesting an existence of the common pathways involved in induction of apoptosis. By molecular network analysis, we identified the ErbB (EGFR) signaling pathway, in addition to axonal guidance and cell adhesion pathways, as those of the significant pathways in downregulated genes shared among all the compounds. Importantly, a recent study by combining genome-wide screening of altered genes and molecular network analysis identified axonal guidance and cell adhesion pathways as those enriched in a variety of cellular processes deregulated in human glioblastoma [18]. We found that the ErbB (EGFR) signaling pathway is composed of FAK/PTK2, AKT3, and GSK3B, serving as key molecules involved in cell movement and the nervous system development. In contrast, the set of upregulated genes by the compounds 1a and 3 showed the most significant relationship with the cell cycle pathway, where CDC25A acts as a hub molecule. Finally, we found that a GSK3B-specific inhibitor induced apoptosis of U373MG cells, supporting an anti-apoptotic role of GSK3B. These observations indicate that molecular network analysis is a useful approach not only to characterize the glioma-relevant pathways but also to identify the network-based effective drug targets.

Being consistent with our observations, downregulation of AKT3 expression by RNA interference reduces the expression of the phosphorylated form of Bad, resulting in induction of the caspase-dependent apoptosis of glioma cells [19]. AKT3 is required for anchorage-independent growth of human glioma cells [20]. GSK3 is a serine/threonine kinase that regulates in an integrated manner Wnt/β-catenin, Hedgehog, and receptor tyrosine kinase (RTK) signaling pathways involved in a wide range of cellular functions, such as glycogen metabolism, cell differentiation, proliferation, and apoptosis [21]. Interference with GSK3β activity by siRNA inhibits cell migration and induces apoptosis of glioma cells by activating c-Myc and inactivating nuclear factor-κB (NF-κB) activities [22,23].

The cell cycle progression is positively and negatively regulated by the complex checkpoint mechanism that involves cyclins A, B, D, and E, along with cyclin-dependent kinases (CDKs) and CDK inhibitors (CDKIs) of both the Cip/Kip and Ink4 families. The hypophosphorylated Rb protein interacts with the E2F family transcription factors E2F1, E2F2, and E2F3, and activates the expression of the genes pivotal for cell cycle progression, whereas the Rb protein, hyperphosphorylated by cyclin D1-CDK4 and cyclin E1-CDK2 complexes, releases E2Fs, and represses the expression of cell cycle genes [24]. Thus, the Rb/E2F pathway constitutes a molecular switch deciding either progression or arrest of the cell cycle. By using KeyMolnet, we found that the molecular network, composed of the combined set of downregulated and upregulated genes in U373MG cells shared between the compounds 1a and 3, shows the most significant relationship with transcriptional regulation by Rb/E2F transcription factors. These observations suggest that both of the structurally-related tetrahydroisoquinoline alkaloids act as a DNA-alkylating agent that interferes with cell division, leading to apoptosis of target cells via the mechanisms very similar to those of ET-743-mediated cytotoxicity [7].

CDC25A encodes a protein phosphatase that activates cyclin/CDK complexes by removing inhibitory phosphates from the conserved threonine and tyrosine residues on CDK proteins. A previous study showed that inhibition of CDC25A expression induces apoptosis of human glioma cells [25], seemingly contradictory to upregulation of CDC25A during induction of apoptosis of U373MG cells in our study. However, a recent study showed that CDC25A, by activating cyclin B1 and Cdc2, acts as a proapoptotic mediator that enhances staurosporine-induced apoptosis, supporting our observations [26].

We identified ELMO1 as one of top 10 downregulated genes and OSR2 as one of top 10 upregulated genes in U373MG cells exposed to all three compounds. Importantly, ELMO1, by forming the complex with dedicator of cytokinesis 180 (Dock180), serves as a Rac1 guanine nucleotide exchange factor that stimulates glioma cell migration and invasion [27]. OSR2 acts as a transcription factor for expression of the genes involved in the epithelial and mesenchymal interaction during craniofacial development and cellular differentiation [28].

## Conclusion

Molecular network analysis suggested that novel marine-derived anti-cancer 1,2,3,4-tetrahydroisoquinoline alkaloids 1a, 2a, and 3 induced apoptosis of glioma cells through the shared molecular mechanisms involving multiple pathways and targets that play a pivotal role in the survival and invasion of glioma cells.

## Competing interests

The authors declare that they have no competing interests.

## Authors’ contribution

HT carried out the molecular biological experiments. JS analyzed the microarray data and drafted the manuscript. NS, KS, and KC prepared 1,2,3,4-tetrahydroisoquinolines 1a, 2a, and 3. All authors have read and approved the final manuscript.

## Supplementary Material

Additional file 1 The principal component analysis of RMA-normalized microarray data. Click here for file

Additional file 2: Table S1 The set of 426 genes downregulated in U373MG cells following exposure to ET-770 (the compound 1a). Click here for file

Additional file 3: Table S2 The set of 45 genes upregulated in U373MG cells following exposure to ET-770 (the compound 1a). Click here for file

Additional file 4: Table S3 The set of 417 genes downregulated in U373MG cells following exposure to ET-770 derivative (the compound 3). Click here for file

Additional file 5: Table S4 The set of 84 genes upregulated in U373MG cells following exposure to ET-770 derivative (the compound 3). Click here for file

Additional file 6: Table S5 The set of 274 genes downregulated in U373MG cells following exposure to RM (the compound 2a). Click here for file

Additional file 8: Table S7 The set of 196 downregulated genes and 6 upregulated genes in U373MG cells shared among the compounds 1a, 2a, and 3 treatments. Click here for file

Additional file 7: Table S6 The set of 9 genes upregulated in U373MG cells following exposure to RM (the compound 2a). Click here for file

Additional file 9 The PANTHER molecular network of downregulated genes in U373MG cells following exposure to ET-770, the compound 1a. Click here for file

Additional file 10 The IF analysis of molecular network of downregulated genes in U373MG cells following exposure to ET-770, the compound 1a. Click here for file

## References

[B1] Ohgaki H Kleihues P Genetic alterations and signaling pathways in the evolution of gliomas Cancer Sci 2009 100 2235 2241 10.1111/j.1349-7006.2009.01308.x 19737147 PMC11159448

[B2] Li B Yuan M Kim IA Chang CM Bernhard EJ Shu HK Mutant epidermal growth factor receptor displays increased signaling through the phosphatidylinositol-3 kinase/AKT pathway and promotes radioresistance in cells of astrocytic origin Oncogene 2004 23 4594 4602 10.1038/sj.onc.1207602 15077177

[B3] Clarke J Butowski N Chang S Recent advances in therapy for glioblastoma Arch Neurol 2010 67 279 283 10.1001/archneurol.2010.5 20212224

[B4] Satoh J Tabunoki H Arima K Molecular network analysis suggests aberrant CREB-mediated gene regulation in the Alzheimer disease hippocampus Dis Markers 2009 27 239 252 20037212 10.3233/DMA-2009-0670 PMC3835274

[B5] Albert R Jeong H Barabasi AL Error and attack tolerance of complex networks Nature 2000 406 378 382 10.1038/35019019 10935628

[B6] Garcia-Carbonero R Supko JG Manola J Seiden MV Harmon D Ryan DP Quigley MT Merriam P Canniff J Goss G Matulonis U Maki RG Lopez T Puchalski TA Sancho MA Gomez J Guzman C Jimeno J Demetri GD Phase II and pharmacokinetic study of ecteinascidin 743 in patients with progressive sarcomas of soft tissues refractory to chemotherapy J Clin Oncol 2004 22 1480 1490 10.1200/JCO.2004.02.098 15084621

[B7] Gajate C An F Mollinedo F Differential cytostatic and apoptotic effects of ecteinascidin-743 in cancer cells Transcription-dependent cell cycle arrest and transcription-independent JNK and mitochondrial mediated apoptosis. J Biol Chem 2002 277 41580 41589 10.1074/jbc.M204644200 12198119

[B8] Martínez N Sánchez-Beato M Carnero A Moneo V Tercero JC Fernández I Navarrete M Jimeno J Piris MA Transcriptional signature of ecteinascidin 743 (yondelis, trabectedin) in human sarcoma cells explanted from chemo-naive patients Mol Cancer Ther 2005 4 814 823 10.1158/1535-7163.MCT-04-0316 15897246

[B9] D’Incalci M Galmarini CM A review of trabectedin (ET-743): a unique mechanism of action Mol Cancer Ther 2010 9 2157 2163 10.1158/1535-7163.MCT-10-0263 20647340

[B10] Suwanborirux K Charupant K Amnuoypol S Pummangura S Kubo A Saito N Ecteinascidins 770 and 786 from the Thai tunicate Ecteinascidia thurstoni J Nat Prod 2002 65 935 937 10.1021/np010485k 12088444

[B11] Saktrakulkla P Toriumi S Tsujimoto M Patarapanich C Suwanborirux K Saito N Chemistry of ecteinascidins. Part 3: preparation of 2’-N-acyl derivatives of ecteinascidin 770 and evaluation of cytotoxicity Bioorg Med Chem 2011 19 4421 4436 10.1016/j.bmc.2011.06.047 21752654

[B12] Suwanborirux K Amnuoypol S Plubrukarn A Pummangura S Kubo A Tanaka C Saito N Chemistry of renieramycins. Part 3: isolation and structure of stabilized renieramycin type derivatives possessing antitumor activity from Thai sponge Xestospongia species, pretreated with potassium cyanide J Nat Prod 2003 66 1441 1446 10.1021/np030262p 14640515

[B13] Charupant K Suwanborirux K Daikuhara N Yokoya M Ushijima-Sugano R Kawai T Owa T Saito N Microarray-based transcriptional profiling of renieramycin M and jorunnamycin C, isolated from Thai marine organisms Mar Drugs 2009 7 483 494 10.3390/md7040483 20098592 PMC2810219

[B14] Halim H Chunhacha P Suwanborirux K Chanvorachote P Anticancer and antimetastatic activities of renieramycin M, a marine tetrahydroisoquinoline alkaloid, in human non-small cell lung cancer cells Anticancer Res 2011 31 193 202 21273598

[B15] Charupant K Daikuhara N Saito E Amnuoypol S Suwanborirux K Owa T Saito N Chemistry of renieramycins. Part 8: synthesis and cytotoxicity evaluation of renieramycin M-jorunnamycin A analogues Bioorg Med Chem 2009 17 4548 4558 10.1016/j.bmc.2009.05.009 19457672

[B16] da Huang W Sherman BT Lempicki RA Systematic and integrative analysis of large gene lists using DAVID bioinformatics resources Nat Protoc 2009 4 44 57 19131956 10.1038/nprot.2008.211

[B17] Draghici S Khatri P Tarca AL Amin K Done A Voichita C Georgescu C Romero R A systems biology approach for pathway level analysis Genome Res 2007 17 1537 1545 10.1101/gr.6202607 17785539 PMC1987343

[B18] Parsons DW Jones S Zhang X Lin JC Leary RJ Angenendt P Mankoo P Carter H Siu IM Gallia GL Olivi A McLendon R Rasheed BA Keir S Nikolskaya T Nikolsky Y Busam DA Tekleab H Diaz LA Hartigan J Smith DR Strausberg RL Marie SK Shinjo SM Yan H Riggins GJ Bigner DD Karchin R Papadopoulos N Parmigiani G Vogelstein B Velculescu VE Kinzler KW An integrated genomic analysis of human glioblastoma multiforme Science 2008 321 1807 1812 10.1126/science.1164382 18772396 PMC2820389

[B19] Mure H Matsuzaki K Kitazato KT Mizobuchi Y Kuwayama K Kageji T Nagahiro S Akt2 and Akt3 play a pivotal role in malignant gliomas Neuro Oncol 2010 12 221 232 10.1093/neuonc/nop026 20167810 PMC2940586

[B20] Endersby R Zhu X Hay N Ellison DW Baker SJ Nonredundant functions for Akt isoforms in astrocyte growth and gliomagenesis in an orthotopic transplantation model Cancer Res 2011 71 4106 4116 10.1158/0008-5472.CAN-10-3597 21507933 PMC3118569

[B21] Doble BW Woodgett JR GSK-3: tricks of the trade for a multi-tasking kinase J Cell Sci 2003 116 1175 1186 10.1242/jcs.00384 12615961 PMC3006448

[B22] Kotliarova S Pastorino S Kovell LC Kotliarov Y Song H Zhang W Bailey R Maric D Zenklusen JC Lee J Fine HA Glycogen synthase kinase-3 inhibition induces glioma cell death through c-MYC, nuclear factor-kB, and glucose regulation Cancer Res 2008 68 6643 6651 10.1158/0008-5472.CAN-08-0850 18701488 PMC2585745

[B23] Korur S Huber RM Sivasankaran B Petrich M Morin P Hemmings BA Merlo A Lino MM GSK3β regulates differentiation and growth arrest in glioblastoma PLoS One 2009 4 7443 10.1371/journal.pone.0007443 PMC2757722 19823589

[B24] Swiss VA Casaccia P Cell-context specific role of the E2F/Rb pathway in development and disease Glia 2010 58 377 390 19795505 10.1002/glia.20933 PMC2882865

[B25] Yamashita Y Kasugai I Sato M Tanuma N Sato I Nomura M Yamashita K Sonoda Y Kumabe T Tominaga T Katakura R Shima H CDC25A mRNA levels significantly correlate with Ki-67 expression in human glioma samples J Neurooncol 2010 100 43 49 10.1007/s11060-010-0147-3 20217459

[B26] Chou ST Yen YC Lee CM Chen MS Pro-apoptotic role of Cdc25A: activation of cyclin B1/Cdc2 by the Cdc25A C-terminal domain J Biol Chem 2010 285 17833 17845 10.1074/jbc.M109.078386 20368335 PMC2878547

[B27] Jarzynka MJ Hu B Hui KM Bar-Joseph I Gu W Hirose T Haney LB Ravichandran KS Nishikawa R Cheng SY ELMO1 and Dock180, a bipartite Rac1 guanine nucleotide exchange factor, promote human glioma cell invasion Cancer Res 2007 67 7203 7211 10.1158/0008-5472.CAN-07-0473 17671188 PMC2867339

[B28] Lan Y Kingsley PD Cho ES Jiang R Osr2, a new mouse gene related to Drosophila odd-skipped, exhibits dynamic expression patterns during craniofacial, limb, and kidney development Mech Dev 2001 107 175 179 10.1016/S0925-4773(01)00457-9 11520675

