# Supplementary material for: Molecular network profiling of U373MG human glioblastoma cells following induction of apoptosis by novel marine-derived anti-cancer 1,2,3,4-tetrahydroisoquinoline alkaloids
Source: Cancer Cell Int. 2012 Apr 11;12:14. doi: 10.1186/1475-2867-12-14 (PMC3441782; doi:10.1186/1475-2867-12-14)
Supplement: Additional file 1 — The principal component analysis of RMA-normalized microarray data. [file 1475-2867-12-14-S1.ppt]

## Slide 1
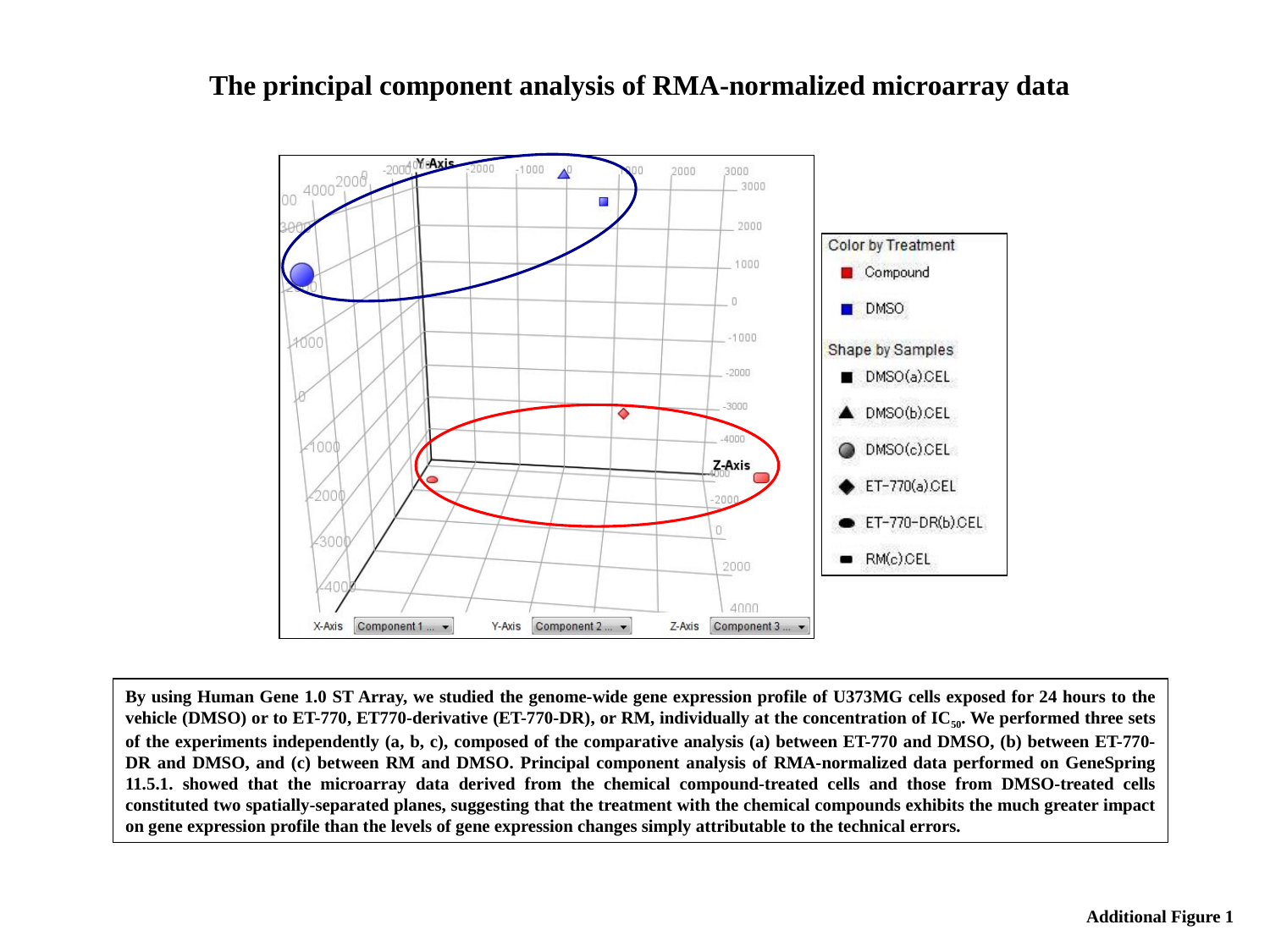

The principal component analysis of RMA-normalized microarray data
By using Human Gene 1.0 ST Array, we studied the genome-wide gene expression profile of U373MG cells exposed for 24 hours to the vehicle (DMSO) or to ET-770, ET770-derivative (ET-770-DR), or RM, individually at the concentration of IC50. We performed three sets of the experiments independently (a, b, c), composed of the comparative analysis (a) between ET-770 and DMSO, (b) between ET-770-DR and DMSO, and (c) between RM and DMSO. Principal component analysis of RMA-normalized data performed on GeneSpring 11.5.1. showed that the microarray data derived from the chemical compound-treated cells and those from DMSO-treated cells constituted two spatially-separated planes, suggesting that the treatment with the chemical compounds exhibits the much greater impact on gene expression profile than the levels of gene expression changes simply attributable to the technical errors.
Additional Figure 1
