# Supplementary material for: Molecular network profiling of U373MG human glioblastoma cells following induction of apoptosis by novel marine-derived anti-cancer 1,2,3,4-tetrahydroisoquinoline alkaloids
Source: Cancer Cell Int. 2012 Apr 11;12:14. doi: 10.1186/1475-2867-12-14 (PMC3441782; doi:10.1186/1475-2867-12-14)
Supplement: Additional file 9 — The PANTHER molecular network of downregulated genes in U373MG cells following exposure to ET-770, the compound 1a. [file 1475-2867-12-14-S9.ppt]

## Slide 1
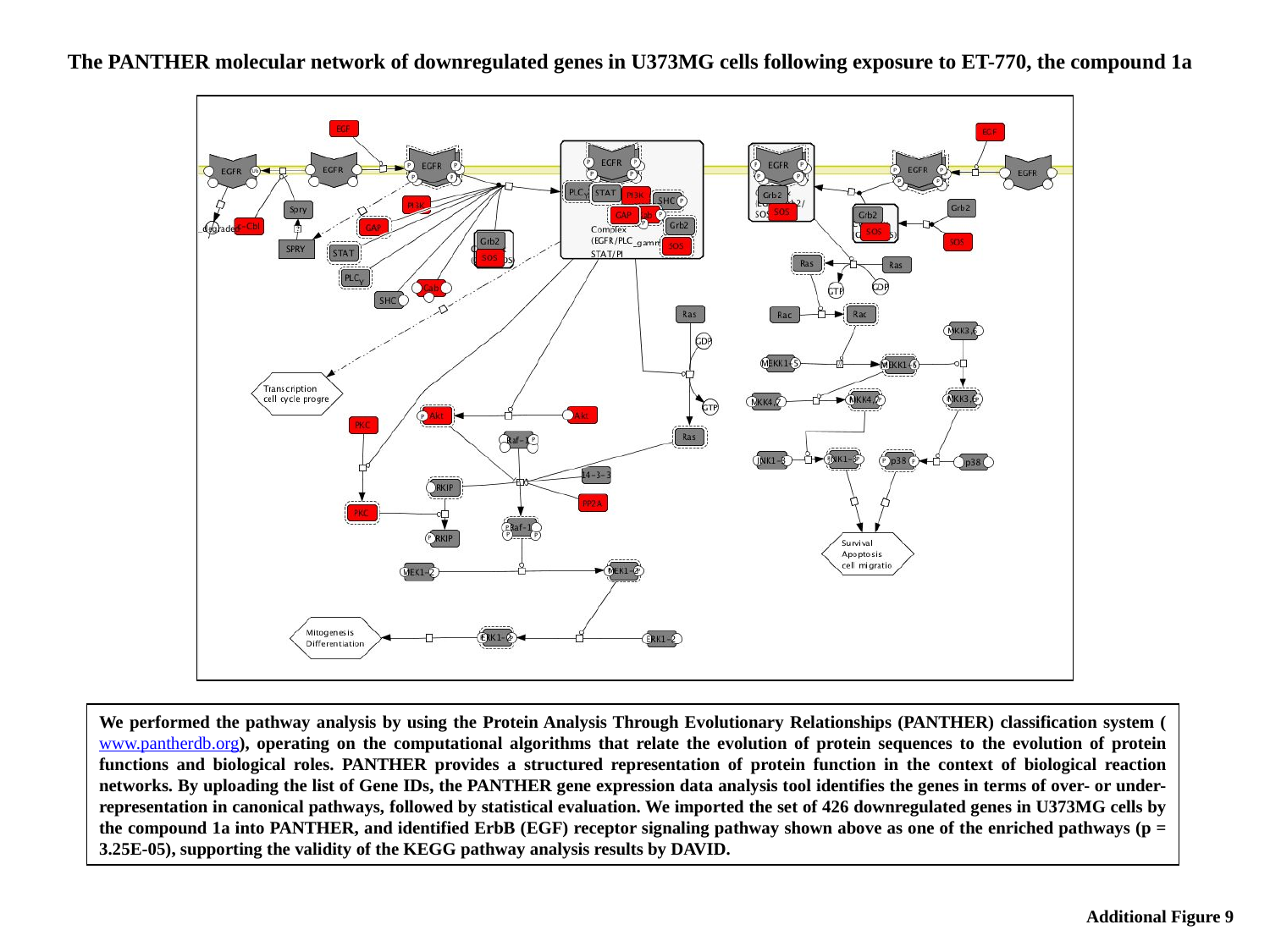

The PANTHER molecular network of downregulated genes in U373MG cells following exposure to ET-770, the compound 1a
We performed the pathway analysis by using the Protein Analysis Through Evolutionary Relationships (PANTHER) classification system (www.pantherdb.org), operating on the computational algorithms that relate the evolution of protein sequences to the evolution of protein functions and biological roles. PANTHER provides a structured representation of protein function in the context of biological reaction networks. By uploading the list of Gene IDs, the PANTHER gene expression data analysis tool identifies the genes in terms of over- or under-representation in canonical pathways, followed by statistical evaluation. We imported the set of 426 downregulated genes in U373MG cells by the compound 1a into PANTHER, and identified ErbB (EGF) receptor signaling pathway shown above as one of the enriched pathways (p = 3.25E-05), supporting the validity of the KEGG pathway analysis results by DAVID.
Additional Figure 9
