# Supplementary material for: Molecular network profiling of U373MG human glioblastoma cells following induction of apoptosis by novel marine-derived anti-cancer 1,2,3,4-tetrahydroisoquinoline alkaloids
Source: Cancer Cell Int. 2012 Apr 11;12:14. doi: 10.1186/1475-2867-12-14 (PMC3441782; doi:10.1186/1475-2867-12-14)
Supplement: Additional file 10 — The IF analysis of molecular network of downregulated genes in U373MG cells following exposure to ET-770, the compound 1a. [file 1475-2867-12-14-S10.ppt]

## Slide 1
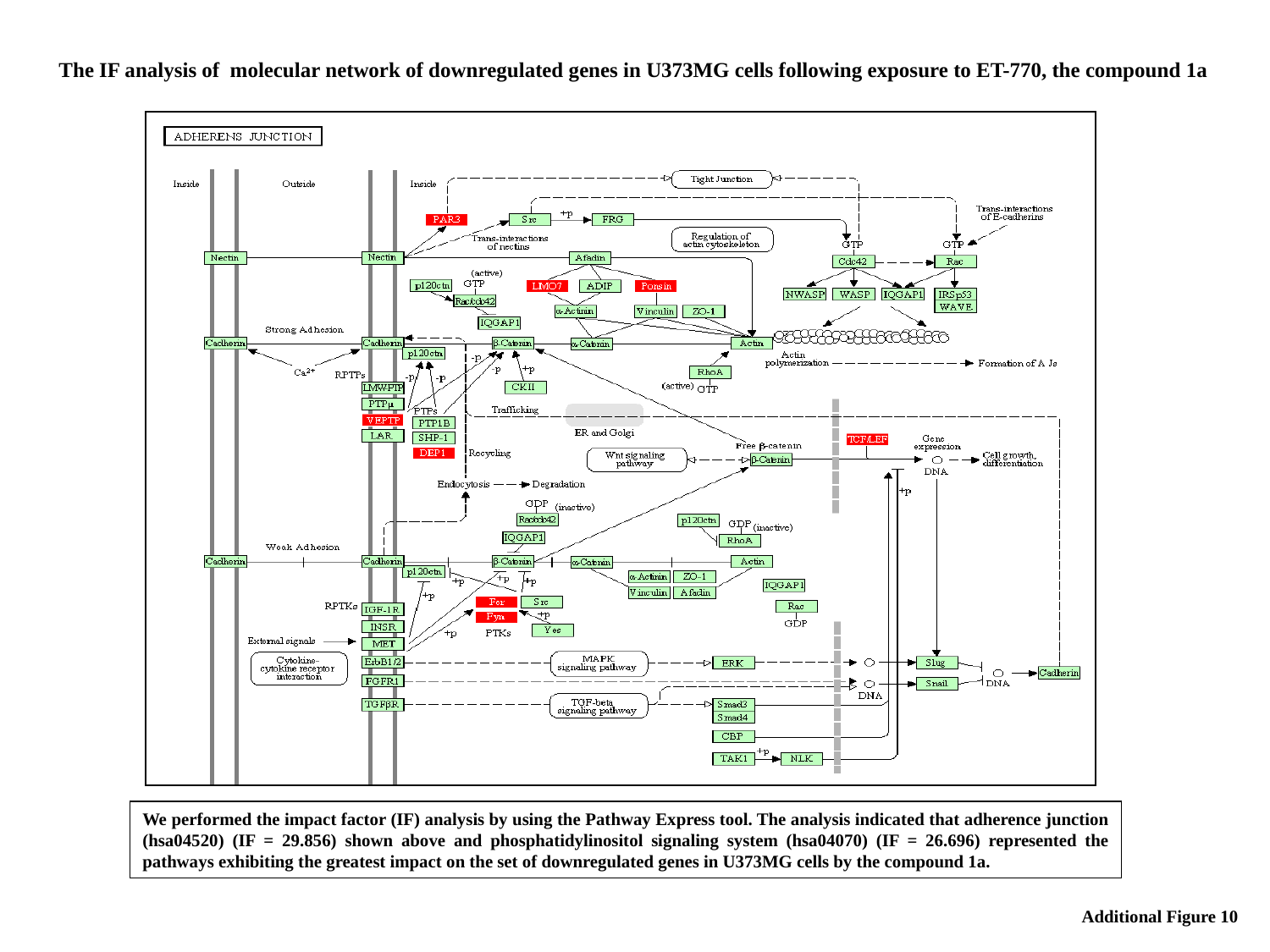

The IF analysis of molecular network of downregulated genes in U373MG cells following exposure to ET-770, the compound 1a
We performed the impact factor (IF) analysis by using the Pathway Express tool. The analysis indicated that adherence junction (hsa04520) (IF = 29.856) shown above and phosphatidylinositol signaling system (hsa04070) (IF = 26.696) represented the pathways exhibiting the greatest impact on the set of downregulated genes in U373MG cells by the compound 1a.
Additional Figure 10
